# Spatial-Dependent Spectral Response of Acousto-Optic Tunable Filters with Inhomogeneous Acoustic Distribution

**DOI:** 10.3390/ma17184537

**Published:** 2024-09-15

**Authors:** Shujing Sun, Huijie Zhao, Qi Guo, Yijie Wang

**Affiliations:** 1 School of Instrumentation and Opto-Electronic Engineering, Beihang University, Beijing 100191, China; sunshujing@buaa.edu.cn (S.S.);; 2 Qingdao Research Institute of Beihang University, Qingdao 266104, China; 3 Institute of Artificial Intelligence, Beihang University, Beijing 100191, China; 4 Aerospace Optical-Microwave Integrated Precision Intelligent Sensing Key Laboratory of Ministry of Industry and Information Technology, Beihang University, Beijing 100191, China

**Keywords:** AOTF, inhomogeneous acoustic distribution, acousto-optic interaction, spatial-spectral response

## Abstract

The spectral response of an acousto-optic tunable filter (AOTF) is crucial for an AOTF based spectral imaging system. The acousto-optic (AO) interaction within the spatial-distributed area of the acoustic field determines the spectral response of the light incidence. Assuming an ideally uniform acoustic field distribution, phase-matching geometries can be applied to calculate the anisotropic Bragg diffraction in AO interactions, determining the wavelength and direction of the diffracted light. In this ideal scenario, the wavelength of the diffracted light depends solely on the direction of the incident light. However, due to the non-ideal nature of the acoustic field, the wavelength of the diffracted light exhibits slight variations with incident position. In this paper, an analytical model is proposed to calculate the spatial-dependent spectral response of the diffracted light under non-uniform acoustic field distribution. The study computes the variation pattern of the diffracted light amplitude caused by the inhomogeneous acoustic distribution. The theoretical considerations and computational model are confirmed by AOTF frequency scanning experiments. The study demonstrates that the distribution of the acoustic field leads to non-uniform spatial-spectral response in the AOTF, and the spatial AO interaction computational model can provide data support for calibrating AOTF systems in imaging applications.

## 1. Introduction

Acousto-optic tunable filters (AOTFs) have gained considerable attention in recent years as spectral filtering devices for imaging spectrometers due to their advantages, including rapid band-switching, continuous wavelength tuning, flexible switching capabilities, and the absence of mechanical moving parts [1,2,3,4]. As AOTF is a bulk acoustic wave diffraction device, the aberration and spectral distribution characteristics of a spectral imaging system utilizing AOTF as the core spectral filtering element differ significantly from those of traditional filter-based spectral imaging systems. For instance, in the collimating (telescopic) scheme, spectral images obtained using AOTF exhibit asymmetric distortion in the diffractive direction due to the angular-dependent spatial characteristics [5], along with spectral inhomogeneity across the field of view caused by angular-dependent spectral characteristics [6]. Therefore, an accurate description of the spectral filtering characteristics of AOTF is crucial for the image quality analysis of spectral imaging systems. Currently, the plane-wave approximation of acoustic field is typically applied in analyzing AOTF for spectral imaging. Under this approximation, the acoustic wave is considered a sinusoidal wave, with the direction of acoustic phase velocity perpendicular to the transducer surface [7,8] and acoustic energy velocity along the direction of acoustic walk-off [9,10,11]. Since the size of the AOTF transducer cannot be infinitely large, the acoustic field distribution within the AOTF differs from that of a plane wave [12,13,14]. Additionally, the acoustic anisotropy of acousto-optic crystals such as LiNbO_3_ and TeO_2_ also affects the degree of non-uniformity in the acoustic field distribution [15]. In the actual application process, the diffraction presents an inhomogeneous energy distribution due to the fact that the acoustic field deviates from the plane wave [16]. Thus, for analyzing the influence of the AOTF’s spatial and spectral response on imaging using the AO interaction, the spatial distribution of the acoustic field cannot be ignored.

Currently, the plane wave angular spectrum method is widely used for the simulation of the acoustic field distribution within an AOTF device [15,17]. And then, the AO interaction equation derived from the Raman-Nath equations allows for numerical analysis of the Bragg’s acousto-optic diffraction [18,19,20,21]. Many reported articles use this method to analyze the AO diffraction [22,23]. However, most of the works were concentrated on the decrease or even increase in diffraction efficiency [14,22]. The shift of the spectral response of AOTF due to the inhomogeneous distribution of the amplitude and angle of the acoustic field lacks investigation.

In this paper, to determine the impact of spatial-dependent spectral response caused by non-uniform acoustic field distribution on AOTF imaging, a three-dimensional spatial AO interaction computational model was realized to simulate the AO diffraction at different incident conditions. The computational model was confirmed by the experiments on AOTF frequency sweeping experiments, thereby calculating the distribution of the center wavelength of the optical aperture under fixed spatial incident angle conditions with collimated light.

## 2. Methods

**Figure 1:**
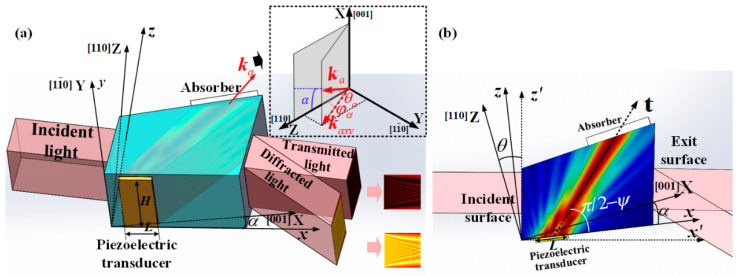
(**a**) The schematic diagram of AOTF acousto-optic diffraction; (**b**) Schematic diagram of the AOTF XZ plane.

A volume grating is formed in the AO crystal due to the shear acoustic wave traveled inside, which is generated from the vacuum-bonded transducer and absorbed by the sound absorber, as shown in Figure 1a. In the schematic diagram of the AOTF device, we use X, Y, and Z to represent the crystal coordinate [001] axis, $\left[1\bar{1}0\right]$ axis, and [110] axis, respectively. The *x*, y, and z axes correspond to the transducer’s coordinate system, with the y-axis coinciding with the Y-axis. The x-axis lies within the transducer plane, forming the transducer cutting angle α with the X-axis. The transducer with a length of L and a width of H is bonded on the xy plane. Under ideal acoustic plane-wave assumption, the acoustic field with phase velocity perpendicular to the transducer propagates along the group velocity direction at the transducer’s vibration frequency *f* and has a uniformly distributed intensity. The t-axis represents the direction of acoustic energy propagation, with a deviation angle of walk-off angle ψ from the phase velocity direction. The walk-off angle ψ is determined by the phase velocity direction and the slowness ellipsoid [11].

However, due to the finite size of the transducer and crystal, the acoustic field within the AOTF device exhibits non-uniformity, represented by the direction of the acoustic wave vector $k_{a}(x,y,z)$ having an inhomogeneous distribution. Additionally, apart from the direction, the length of the acoustic wave vector $k_{a}(x,y,z)$ = *2πf*/*v* also varies, since the phase velocity $v(\theta_{\alpha },\varphi_{\alpha })$ depends on the phase propagating direction due to the acoustic anisotropy of the TeO_2_ crystal. The upper-right insertion in Figure 1a shows the wave vector $k_{a}$ in the crystal coordinate system, where $k_{axy}$ is the projection of $k_{a}$ on the YZ plane, $\theta_{\alpha }$ is the acoustic polar angle between $k_{a}$ and $k_{axy}$, and $\varphi_{\alpha }$ is the acoustic azimuth angle between $k_{axy}$ and the *Z*-axis. Figure 1b shows a schematic diagram of the acoustic path on the XZ plane, which is also defined as the AO interaction plane of the AOTF device. When the light is perpendicularly incident onto the AOTF optical aperture, the coordinate x′-axis follows the incident orientation. The angle between the z′-axis and the *Z*-axis in Figure 1b is θ, where θ is the incident plane cut angle.

Assuming that the AO crystal is a linear uniform medium, the acoustic perturbation ${u_{0}(x,y)|}_{z=0}$ generated by the transducer at the plane *z* = 0 is expanded into a plane angular spectrum $U(k_{ax},k_{ay})$ by using the spatial Fourier transform.

(1) $$U_{0}(k_{ax},k_{ay})=\iint_{\infty }u_{0}(x,y)\exp[i(k_{ax}x+k_{ay}y)]dxdy$$

Here, $k_{ax},k_{ay}$ are the components of the acoustic wave vector $k_{a}$ in the x, y axes, respectively. Through numerical integration, the acoustic field propagating with a distance of *z* in the AO crystal can be obtained [16,24].

(2) $$u(x,y,z)=\left|u(x,y,z)\right|e^{i\Phi }=f^{2}\times \iint_{\infty }U(k_{ax},k_{ay},z)\exp[-i(k_{ax}x+k_{ay}y)]dk_{ax}dk_{ay}$$

Here, f is the ultrasonic driving frequency. The |u(x,y,z)| is the acoustic field amplitude distribution. The arg(u(x,y,z)) is the acoustic field phase distribution. The gradient of the phase Φ in different directions can be used to determine the acoustic polar angle $\theta_{\alpha }$ and acoustic azimuth angle $\varphi_{\alpha }$. $U(k_{ax},k_{ay},z)$ represents the angular spectrum distribution of the spatial acoustic field.

(3) $$U(k_{ax},k_{ay},z)=U_{0}(k_{ax},k_{ay})\exp[-iz\sqrt{k_{a}^{2}-k_{ax}^{2}-k_{ay}^{2}}]$$

**Figure 2:**
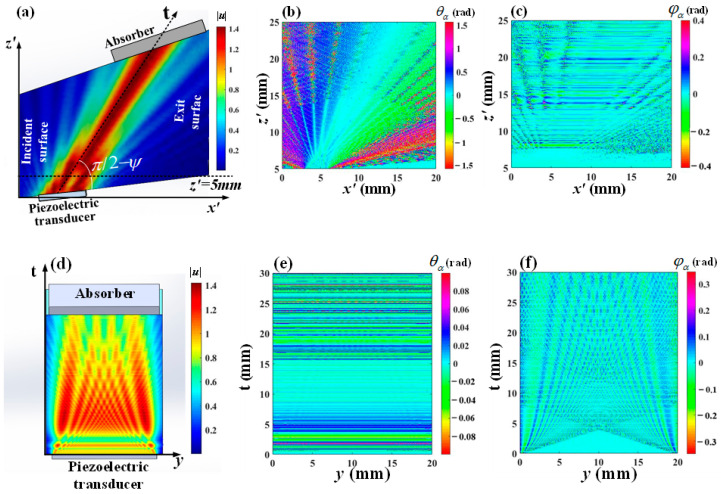
Amplitude and angle distribution of the acoustic field in different cross-sections (**a**) Amplitude distribution of the acoustic field on the x′z′ plane; (**b**) Acoustic polar angle distribution of the acoustic field on the x′z′ plane; (**c**) Acoustic azimuth angle distribution of the acoustic field on the x′z′ plane; (**d**) Amplitude distribution of the acoustic field on the yt plane; (**e**) Acoustic polar angle distribution of the acoustic field on the yt plane; (**f**) Acoustic azimuth angle distribution of the acoustic field on the yt plane.

To analyze the acoustic field distribution within the AOTF, a simulation was conducted using the existing AOTF parameters available in the laboratory. The size of the optical aperture is set to 20 mm × 20 mm, the transducer cutting angle α is 6.5°, and the transducer size is 3 mm × 20 mm, where L and H are 3 mm and 20 mm, respectively. Figure 2a shows the acoustic amplitude distribution of the AOTF on the y = H/2 plane. Figure 2b and 2c, respectively, show the distributions of the acoustic polar angle $\theta_{\alpha }$ and acoustic azimuth angle $\varphi_{\alpha }$ on the plane y = H/2. The range of the horizontal axis x′ is from 5 mm to 25 mm, with the vertical axis corresponding to the dashed line at z′ = 5 mm in Figure 2a. Figure 2d shows the acoustic amplitude distribution of the AOTF on the *y*t plane. Figure 2e and Figure 2f, respectively, show the distributions of the acoustic polar angle $\theta_{\alpha }$ and acoustic azimuth angle $\varphi_{\alpha }$ in the *y*t plane.

Through the acoustic field distribution on the AO interaction plane shown in Figure 2a–c, it is evident that the amplitude distribution of the acoustic field diverges significantly with increasing propagation distance, while the acoustic polar angle distribution ranges approximately from −1.6 to 1.5, and the acoustic azimuth angle distribution ranges from −0.4 to 0.4. Therefore, in the analysis of AO interactions, it is necessary to comprehensively consider the amplitude, polar angle, and azimuth angle of the acoustic field. Through the acoustic field distribution in the *y*t plane shown in Figure 2d–f, it is observed that the amplitude of the acoustic field exhibits a divergent distribution, and the main lobe energy can cover the entire *y*t plane, with the acoustic polar angle ranging from −0.1 to 0.1 and the acoustic azimuth angle ranging from −0.4 to 0.4. Notably, the distribution of the acoustic amplitude is similar to that of the acoustic azimuth angle. 

To solve the diffracted light resulting from the AO interaction, the method of coupled waves is employed and consider the refractive index in the region of AO interaction as a static index with an additional small perturbation Δn due to the photoelastic effect [25].

(4) $$\Delta n=-\frac{1}{2}{n_{m}}^{3}pS$$

Here, p is the AO coefficient matrix of the crystal and S is the strain vector obtained by taking the partial derivative of u(x,y,z), $n_{m},m=0,1$, $n_{0}$ is the refractive index of the incident light, and $n_{1}$ is the refractive index of the diffracted light.

The modified Raman-Nath equation is derived to study the AO interaction inside the AOTF [22,23]. In the Bragg regime, the incident light is diffracted into only one order, thus, considering acoustic the spatial inhomogeneous structure, the AO interaction equation is obtained as:

(5) $$\left\{ \begin{array}{l} \frac{dC_{0}}{{dx}^{\prime }}=\frac{q_{0}(x^{\prime },y^{\prime },z^{\prime })}{2\cos\theta_{0}}C_{1}\exp[i(\frac{\Delta kx^{\prime }}{\cos\theta_{1}}-\Phi (x^{\prime },y^{\prime },z^{\prime }))]\\ \frac{dC_{1}}{{dx}^{\prime }}=-\frac{q_{1}(x^{\prime },y^{\prime },z^{\prime })}{2\cos\theta_{1}}C_{0}\exp[-i(\frac{{\Delta kx}^{\prime }}{\cos\theta_{0}}-\Phi (x^{\prime },y^{\prime },z^{\prime }))] \end{array}\right.$$

**Figure 3:**
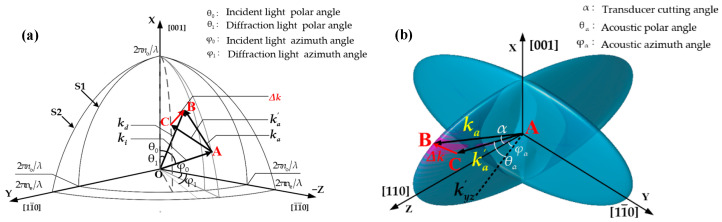
Spatial wave vector diagram in anisotropic crystal. (**a**) Spatial wave vector matching diagram; (**b**) Spatial acoustic wave vector diagram.

Here, $C_{0}$ and $C_{1}$ are the relative amplitudes of the transmitted light and the diffracted light, respectively. Φ represents the acoustic field phase distribution within the AOTF for incident light with a polar angle $\theta_{0}$ and azimuth angle $\varphi_{0}$. As shown in Figure 3a, $\theta_{0}$ is the incident light polar angle between $k_{i}$ and the *Z*-axis in the XZ plane, $\theta_{1}$ is the diffracted light polar angle between $k_{d}$ and the *Z*-axis in the XZ plane. And $\varphi_{0}$ is the azimuth angle of the incident light, defined as the angle between the projection of $k_{i}$ onto the ZY plane and the −Z axis. Similarly, $\varphi_{1}$ is the azimuth angle of the diffraction light, defined as the angle between the projection of $k_{d}$ onto the ZY plane and the −Z axis. $q_{m}$ is the AO coupling coefficient:

(6) $$q_{m}=\frac{2\pi \Delta n}{\lambda \cos\theta_{m}}$$

Here, λ is the wavelength of the incident light. For the case where the incident light is in the extraordinary mode, $n_{0}$, $n_{1}$ are, respectively defined as [26]:

(7) $$\left\{ \begin{array}{l} n_{0}={\left(\cos^{2}\theta_{0}/n_{o}^{2}+\sin^{2}\theta_{0}/n_{e}^{2}\right)}^{-1/2}\\ n_{1}=n_{o} \end{array}\right.$$

Here, $n_{o}$ is the refractive index of the ordinary light of the AO crystal, while $n_{e}$ is the refractive index of the extraordinary light propagating along the [110] axis within the AO crystal.

Figure 3a is a schematic diagram of AO coupling at an arbitrary point. Assume that the acoustic wave vector $k_{a}$ perpendicular to the transducer satisfy the momentum matching with the incident wave vector $k_{i}$ and diffractive wave vector $k_{d}$. However, due to the inhomogeneous distribution of the acoustic field, the wave vector ${k_{a}}^{\prime }$ exists, resulting in the momentum mismatching Δk [27,28]. The acoustic wave vectors $k_{a}$, ${k_{a}}^{\prime }$ and momentum mismatching Δk on the acoustic wave vector surface [29] are shown in Figure 3b. Under ideal acoustic field conditions, only the wave vector $k_{a}$ exists; the endpoint of the acoustic wave vector will only exist at point B on the wave vector surface in Figure 3b, where the acoustic-optic momentum matching is considered to be achieved. However, in a non-uniform acoustic field, the endpoints of the wave vectors will be distributed around point B, with the wave vectors in the surrounding area of point B being in a momentum mismatch state. In this paper, we only consider the influence of the momentum mismatching Δk on the diffracted light intensity, assuming it has no effect on the direction of the diffracted light. Solving Δk in Figure 3 yields

(8) $$\begin{array}{cc} \Delta k(x,y,z) & =k_{a}-k_{a}^{\prime }\\ & =\sqrt{{\left(k_{a}\cos\alpha -k_{a}^{\prime }\cos\theta_{\alpha }\cos\varphi_{\alpha }\right)}^{2}+{\left(k_{a}^{\prime }\cos\theta_{\alpha }\sin\varphi_{\alpha }\right)}^{2}+{\left(k_{a}\sin\alpha -k_{a}^{\prime }\sin\theta_{\alpha }\right)}^{2}} \end{array}$$

The AO interaction shown in Figure 3 represents the AO diffraction at a single point, while the incident light passes through the entire wave XZ plane with an incident polar angle of $\theta_{0}$, azimuth angle of $\varphi_{0}$, so the final diffracted light is the superposition of the AO interaction of all points in the incident light path.

The input parameters involved in this computational model include: driving frequency, incident light wavelength, incident light polar angle, incident light azimuthal angle, transducer size, acousto-optic crystal cutting angle, and the simulation size of the acousto-optic crystal. By computing the model, one can determine the acousto-optic interaction at different positions within the crystal and the diffraction efficiency at various positions of the optical aperture. Under the condition of keeping other input parameters constant, varying the driving frequency allows for obtaining the frequency response of the AOTF imaging. Using this frequency response, the spatial spectral response can also be calculated.

## 3. Results and Discussion

**Figure 4:**
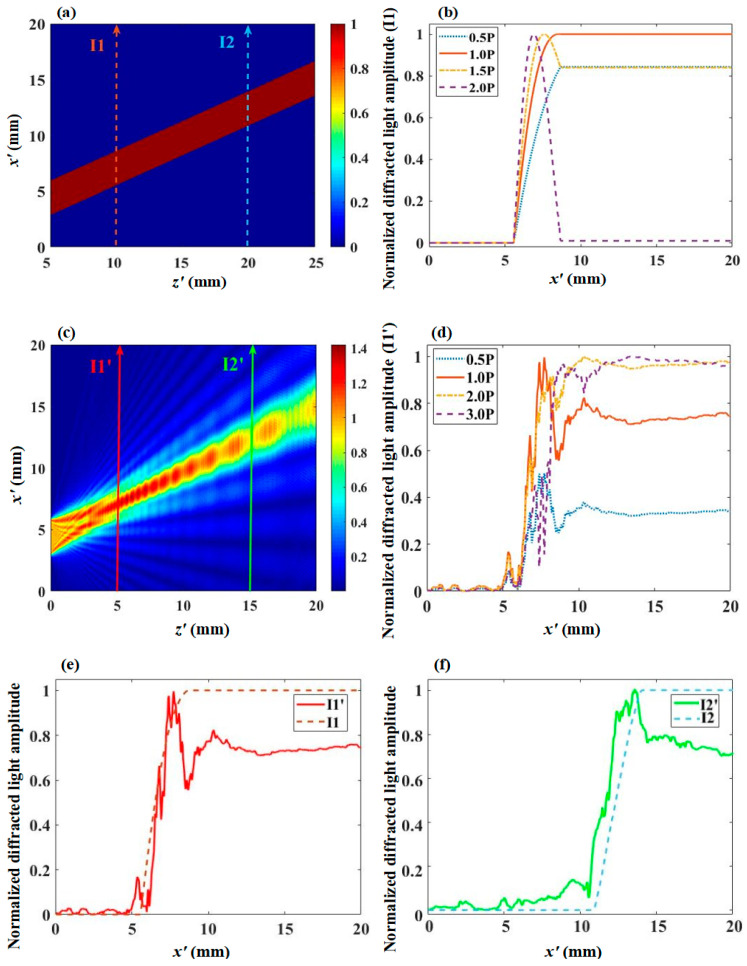
Comparison of ideal acoustic field and simulated acoustic field in x′z′ plane (**a**) Amplitude distribution of ideal acoustic field in x′z′ plane; (**b**) The cumulative variation curve of the amplitude of the diffracted light I1 in the ideal acoustic field under different driving power levels; (**c**) Amplitude distribution of simulated acoustic field in x′z′ plane; (**d**) The cumulative variation curve of the amplitude of the diffracted light I1′ in the simulated acoustic field under different driving power levels; (**e**) Curve of amplitude change of diffracted light for paths I1 and I1′; (**f**) Curve of amplitude change of diffracted light for paths I2 and I2′.

We calculate the diffraction efficiency of incident light with a wavelength of 632.8 nm, an incident polar angle $\theta_{0}$ of 15°, and an azimuth angle $\varphi_{0}$ of 0°. As the incident plane cut angle θ is 15°, the incident light direction is perpendicular to the AOTF incident plane. Under ideal acoustic field conditions, as shown in Figure 4a, the efficiency of the diffracted light does not depend on the position of incidence on the entrance surface once the direction of the incident light is fixed. Figure 4b shows the diffraction light amplitude curve of light path I1 under different acoustic driving power conditions in an ideal acoustic field. When the acoustic drive power is P, the final amplitude value of the diffracted light is 1, and the diffraction efficiency is equal to the square of the normalized amplitude of the diffracted light, resulting in a diffraction efficiency of 100%. In the ideal acoustic field, the diffraction efficiency and the acoustic drive power follow a cosine distribution. Comparing the diffraction efficiencies at 0.5 P, 1.0 P, 1.5 P, and 2.0 P power conditions in Figure 4b, the corresponding efficiencies are 70.7%, 100%, 70.7%, and 0, respectively, all of which match the ideal plane wave calculation model [23].

On the AO interaction plane corresponding to the middle of the transducer with y = H/2, the simulated result of the acoustic amplitude distribution is shown in Figure 4c. Two lines I1′ and I2′ represent the optical paths experienced by the incident light in Figure 4c, and I1 and I2 are positioned the same, respectively, in Figure 4a. Figure 4d shows the diffraction light amplitude curve of light path I1′ under different acoustic driving power conditions in Figure 4c acoustic field. When the acoustic driving power is P, the final efficiency of the diffracted light is 74.7%. Comparing the diffraction efficiencies under the power of 0.5 P, 1.0 P, 2.0 P, and 3.0 P in Figure 4d, the efficiencies are 11.5%, 55.8%, 94.6%, and 94.7%, respectively. Under the power P, the diffraction efficiency does not reach 100%, and as the incident light moves away from the transducer, the diffraction efficiency remains within a certain range as the power increases.

Figure 4e shows the comparison of the diffracted light amplitude curves for light paths I1 and I1′ under the condition of acoustic driving power P. Similarly, Figure 4f shows the comparison of the diffracted light amplitude curves for light paths I2 and I2′ under the condition of acoustic driving power P. The inhomogeneous distribution of the phase and intensity of the acoustic field will cause the AO interaction superposition effects of incident light to be spatially-dependent, so the final amplitudes of the diffracted light of I1′ and I2′ are different, and the normalized diffracted amplitude cannot reach 1, meaning the diffraction efficiency cannot reach 100%. According to Equation (5), the change of the diffraction light amplitude is determined by the exponential term, and $q_{m}$ determines the magnitude of the change. During the AO interaction process, the diffraction light amplitude is complex. When $\left|C_{1}(x_{n+1},y_{n+1},z_{n+1})|-|C_{1}(x_{n},y_{n},z_{n})\right|$ is positive, the diffraction light amplitude increases; otherwise, the diffraction light amplitude decreases, thus reflecting the energy exchange process between the transmitted light and the diffracted light caused by the inhomogeneous acoustic field distribution.

**Figure 5:**
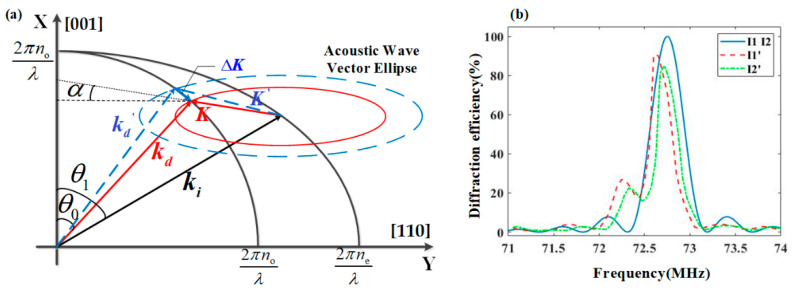
(**a**) Two-dimensional schematic of the influence of acoustic field distribution on the frequency response; (**b**) The frequency response curve between the simulated acoustic field (I1′ I2′) and the ideal acoustic field (I1 I2).

Considering the AO interaction process as the action of the synthetic acoustic wave vector K, the directions of the synthetic acoustic wave vectors depend on the accumulation of the wavevectors through the optical path. Due to the variations in both amplitude and angle of the acoustic field on the XZ plane, Figure 5a provides a schematic illustration of the frequency response of the acoustic field on the XZ plane. As the model calculation does not consider the influence of the acoustic field distribution on the angle of the diffracted light, the wave vectors $K_{i}$ and $K_{d}$ lie on the XZ plane, so the synthetic wave vector K is also on the XZ plane. Due to the different driving frequencies, the acoustic wave vectors K and ${Κ}^{\prime }$ in Figure 5a are in different acoustic wave vector ellipses. At the driving frequency $f_{0}$, the synthesized acoustic wave vector K at a certain position of the optical aperture satisfies the momentum matching. Therefore, $f_{0}$ is the optimal driving frequency at this position.

After changing the driving frequency to $f_{1}$, the synthesized acoustic wave vectors are $K^{\prime }$, which cause vector deviations $\Delta K^{\prime }$ from K. As a result, the diffraction efficiency at the position $K^{\prime }$ is lower than that at the position K, however, the diffraction angle increases with frequency. Through calculations, the optimal drive frequency $f_{0}(x,z)$ at each position of the optical aperture can be obtained. According to the two-dimensional center wavelength calculation formula in [30], the spatial wave vector relationship in Figure 3 can be used to derive the spatial calculation formula for the center wavelength.

(9) $$\lambda =\frac{2\pi }{K}\left\{n_{0}\sin\left(\theta_{0}-\alpha \right)\cos\varphi_{0}-\sqrt{n_{o}^{2}-n_{0}^{2}\left[\cos^{2}\left(\theta_{0}-\alpha \right)+\sin^{2}\left(\theta_{0}-\alpha \right)\sin^{2}\varphi_{0}\right]}\right\}$$

The frequency response curves corresponding to I1, I2, I1′, and I2′ in Figure 4 are presented in Figure 5b. Here, I1 and I2 correspond to ideal acoustic field conditions, where their frequency response curves are identical and symmetrical with the center frequency of 72.75 MHz. In contrast, I1′ and I2′ correspond to non-uniform acoustic field conditions, where their frequency response curves exhibit asymmetrical side lobes. When the diffraction efficiency at the central frequencies of I1 and I2 reaches 100%, the corresponding diffraction efficiencies at the optimal frequencies of I1′ and I2′ are 55.8% and 50.4%, respectively. Due to the non-uniform acoustic field distribution, the center frequencies corresponding to I1′ and I2′ are different from the center frequency under ideal acoustic field conditions.

**Figure 6:**
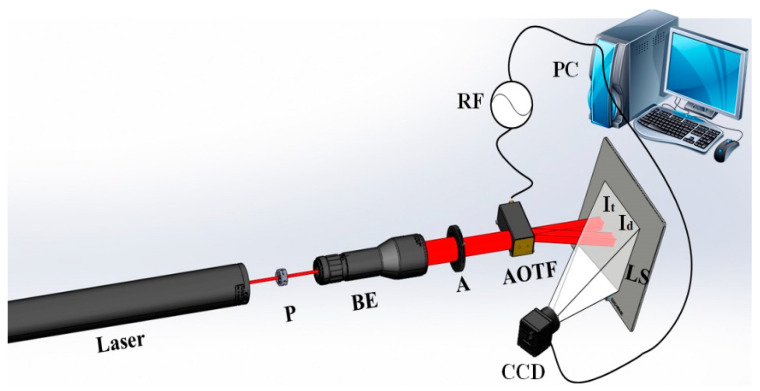
Acoustic-optical diffraction test optical path diagram. P is the polarizer, BE is the beam expander, A is the aperture, RF is the radio frequency signal, I_t_ is the transmitted light, I_d_ is the diffracted light, LS is the light screen, and CCD is the charge coupled device.

In order to verify the method proposed, an optical path is set up as shown in Figure 6 to observe the optimal frequency variation. A 632.8 nm wavelength He-Ne laser (Daheng Optics, Beijing, China) is polarized to extraordinary light by polarizer P (Thorlabs, Newton, NJ, USA). After passing through beam expander BE (Thorlabs, Newton, NJ, USA) and aperture A(Daheng Optics, Beijing, China), a collimated incident light is obtained. After AO diffraction by the AOTF(China Electronics Technology Group Corporation, Chongqing, China), it is divided into transmitted light $I_{t}$ and diffracted light $I_{d}$. The CCD captures the transmitted light and diffracted light on the light screen LS, and the computer PC jointly controls the frequency switching of the AOTF and the data acquisition of the CCD(Basler, Arensburg, Germany).

**Figure 7:**
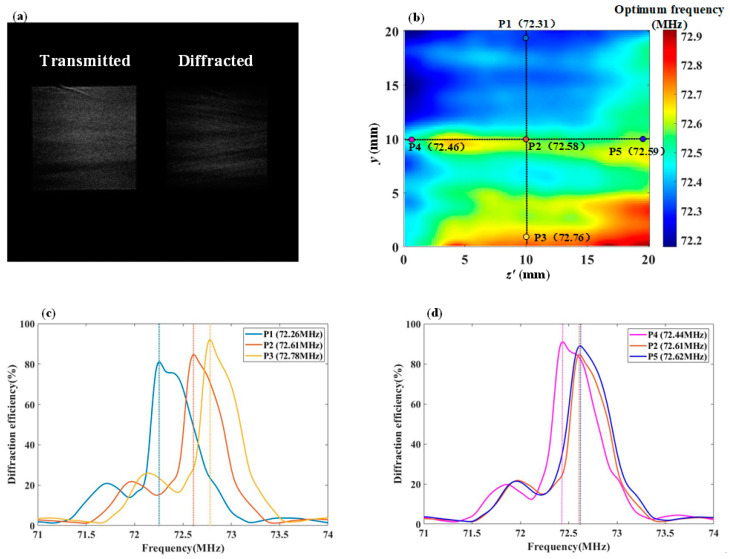
Measured and simulated frequency response data. (**a**) Measured images of transmitted and diffracted light; (**b**) Measured optimal frequency $f_{0}$ across the optical aperture; (**c**) The simulated frequency response curves at P1, P2, and P3,where the dashed line represents the optimal driving frequency position on the frequency curve; (**d**) The simulated frequency response curves at P4, P2, and P5,where the dashed line represents the optimal driving frequency position on the frequency curve.

In order to verify the combined effects of the polar angle and azimuth angle of the incident light, the incident polar angle $\theta_{0}$ is 15°, and the azimuth angle $\varphi_{0}$ is 2°. Figure 7a shows the images of transmitted light and diffracted light captured simultaneously, and the diffraction efficiency is calculated accordingly. The AOTF is swept from 71 MHz to 74 MHz with a frequency interval of 0.01 MHz, and the measured distribution of the optimal driving frequency across the optical aperture is shown in Figure 7b. In Figure 7b, the transducer aligns with the y-axis, and the optimum driving frequency range is 72.17 MHz–72.92 MHz.

Due to the inhomogeneity of the acoustic field distribution, the acoustic field along the path of incident light at different positions varies, resulting in different synthesized acoustic wave vectors $K(y,z^{\prime })$. As a result, the optimal driving frequency across the entire optical aperture is not uniform. The symmetry of the acoustic field distribution is lost, leading to an asymmetric and non-uniform distribution of the optimal driving frequency across the entire optical aperture.

Using the spatial AO interaction calculation method, the frequency response at the five points P1 through P5 in Figure 7b was simulated. In Figure 7b, point P2 is the optical aperture center, while points P1, P3, P4, and P5 are located 8 mm above, below, left, and right of P2, respectively. These points are used to reflect the frequency response differences caused by the non-uniform distribution of the acoustic field. Figure 7c shows the simulated frequency response curves calculated at positions P1, P2, and P3, with the legend indicating the simulated optimal frequencies $f_{0}$. Similarly, Figure 7d shows the simulated frequency response curves calculated at positions P4, P2, and P5.

Table 1 allows for a comparison of the deviation between the measured and simulated values of $f_{0}$ at positions P1 through P5.

By comparing the measured data with the simulated data, it was verified that for the AOTF operating in the visible wavelength range, with a driving frequency between 71 MHz and 74 MHz, the optimal driving frequency $f_{0}$ error within the optical aperture is less than 1%.

When the incident light azimuth angle is 0°, the incident light at positions P1 and P3 experiences the same acoustic field distribution. However, when the incident light has an azimuth angle of 2°, the acoustic field experienced by the incident light at positions P1 and P3 is no longer the same. As shown in Figure 7c, the frequency responses at points P1, P2, and P3 obtained from the computational model differ, and the magnitude of these differences varies with the size and direction of the incident light’s azimuthal angle.

The positions of P4, P2, and P5 reflect the differences in frequency response as the propagation distance of the sound field increases. It is not possible to make the acoustic field distribution experienced by the incident light at different positions the same by changing the incident light angle. As shown in Figure 7d, the optimal driving frequencies at P4, P2, and P5 are similar, and in conjunction with the overall optical aperture distribution shown in Figure 7b, it can be seen that the frequency response differences caused by the incident light azimuthal angle are the primary factor.

Combining the frequency response curves shown in Figure 7c,d, it can be observed that all curves exhibit the phenomenon of asymmetric side lobes. The main reason for this phenomenon is the presence of the incident light polar angle, which causes the acoustic field angles and amplitudes experienced by the incident light to be non-symmetrically distributed, resulting in the frequency response having asymmetric side lobes.

**Figure 8:**
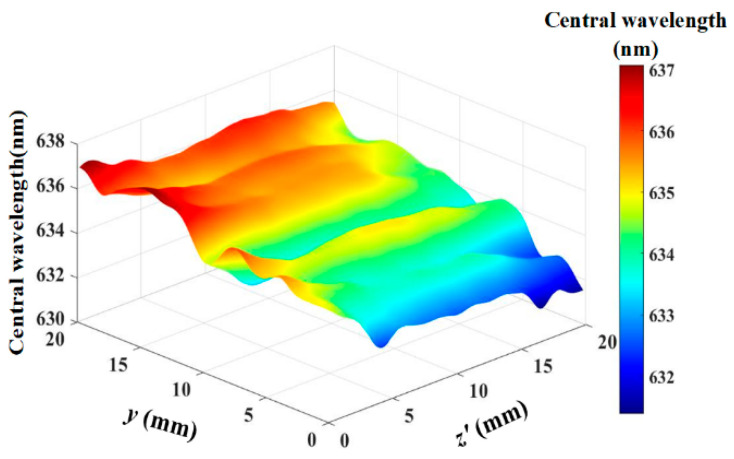
Distribution map of the center wavelength within the optical aperture.

Using Equation (9) to estimate the distribution of the center wavelength within the optical aperture, as shown in Figure 8. The center wavelength distribution ranges from 631.38 nm to 637.07 nm and exhibits an asymmetrical distribution within the optical aperture. This is one of the reasons that the spectral bandwidth of the imaging system is broader than that of the AOTF device.

Currently, the spectral calibration of AOTF is based on the assumption of a uniform distribution of the acoustic field, leading to the belief that the central wavelength of the entire optical aperture is homogeneous. Therefore, the calibration results obtained are only the average values of the tests. However, the spatial spectral distribution data obtained using the computational model presented in this article can improve the data accuracy of the AOTF spectral imaging system. Simultaneously, accurate spectral distribution data can also provide data support for enhancing the spectral resolution in the AOTF imaging system [31,32].

## 4. Conclusions

This paper aims to study the impact of inhomogeneous acoustic field distribution on the spatially-dependent spectral response of AOTF devices. Using the angular spectrum method to calculate the amplitude and angular of the acoustic field within the anisotropic crystal. By combining the AO interaction equation with the acoustic field distribution data, a spatial AO interaction model is obtained. This model allows for the determination of the diffraction efficiency for different incident positions and directions, thereby facilitating the analysis of spatial spectral response. Compared to the current method of transmission function spectral analysis that does not take into account the non-uniform distribution of the acoustic field, this computational model can offer a more accurate analysis of the spectral response of AOTF under various acoustic field distribution conditions.

Due to the inhomogeneous distribution of the acoustic field, the AO interaction process varies at different incident positions under fixed drive frequency conditions, leading to differences in diffraction efficiency. Under the same conditions, the diffraction efficiency of the simulated acoustic field is lower than that of the plane wave acoustic field, indicating that an uneven acoustic field distribution reduces the diffraction efficiency. Under different driving frequency conditions, frequency response data at different positions were obtained using the spatial AO interaction computational model and confirmed by AOTF measured data, with an error of less than 1%. Model calculations indicate that when the spatial angle of the incident light is fixed, the distribution of the center wavelength within the optical aperture is non-uniform. The incident light’s azimuth angle is more sensitive to the non-uniformity of the acoustic field than the polar angle, which can lead to an improvement in the uniformity of the center wavelength distribution. This suggests that in AOTF spectral imaging analysis, it is essential to comprehensively consider the spatial distribution of both the acoustic field and the optical field. This also provides theoretical data support for imaging calibration in AOTF systems.

## Figures and Tables

**Table 1:** Comparison of Measured and Simulated Optimal Driving Frequencies.

Point	$f_{0}\;(MHz)$		Error/%
	Measured	Simulated	
P1	72.31	72.26	0.69
P2	72.58	72.61	0.41
P3	72.76	72.78	0.27
P4	72.46	72.44	0.27
P5	72.59	72.62	0.41

## Data Availability

Data are contained within the article.
